# HIV, antiretroviral treatment, hypertension, and stroke in Malawian adults: A case-control study

**DOI:** 10.1212/WNL.0000000000002278

**Published:** 2016-01-26

**Authors:** Laura A. Benjamin, Elizabeth L. Corbett, Myles D. Connor, Henry Mzinganjira, Sam Kampondeni, Augustine Choko, Mark Hopkins, Hedley C.A. Emsley, Alan Bryer, Brian Faragher, Robert S. Heyderman, Theresa J. Allain, Tom Solomon

**Affiliations:** From the Malawi-Liverpool-Wellcome Trust Clinical Research Programme (L.A.B., E.L.C., A.C., R.S.H.) and the Department of Medicine (L.A.B., H.M., S.K., R.S.H., T.J.A.), College of Medicine, University of Malawi, Blantyre; the Brain Infections Group (L.A.B., T.S.), Institute of Infection and Global Health, University of Liverpool (L.A.B., H.C.A.E., T.S.); the Walton Centre NHS Foundation Trust (L.A.B., T.S.), Liverpool; the Department of Clinical Research (E.L.C.), London School of Hygiene and Tropical Medicine; the NHS Borders and Division of Clinical Neuroscience (M.D.C.), University of Edinburgh, UK; the School of Public Health (M.D.C.), University of the Witwatersrand, Johannesburg, South Africa; the Royal Liverpool Hospital (M.H.); the Royal Preston Hospital (H.C.A.E.), Liverpool, UK; the Department of Medicine (A.B.), Division of Neurology, Groote Schuur Hospital, University of Cape Town, South Africa; the Liverpool School of Tropical Medicine (B.F.); the Division of Infection and Immunity (R.S.H.), University College London; and the National Institute for Health Research (T.S.), Health Protection Research Unit in Emerging and Zoonotic Infections, Liverpool, UK.

## Abstract

**Objective:**

To investigate HIV, its treatment, and hypertension as stroke risk factors in Malawian adults.

**Methods:**

We performed a case-control study of 222 adults with acute stroke, confirmed by MRI in 86%, and 503 population controls, frequency-matched for age, sex, and place of residence, using Global Positioning System for random selection. Multivariate logistic regression models were used for case-control comparisons.

**Results:**

HIV infection (population attributable fraction [PAF] 15%) and hypertension (PAF 46%) were strongly linked to stroke. HIV was the predominant risk factor for young stroke (≤45 years), with a prevalence of 67% and an adjusted odds ratio (aOR) (95% confidence interval) of 5.57 (2.43–12.8) (PAF 42%). There was an increased risk of a stroke in patients with untreated HIV infection (aOR 4.48 [2.44–8.24], *p* < 0.001), but the highest risk was in the first 6 months after starting antiretroviral therapy (ART) (aOR 15.6 [4.21–46.6], *p* < 0.001); this group had a lower median CD4^+^ T-lymphocyte count (92 vs 375 cells/mm^3^, *p* = 0.004). In older participants (HIV prevalence 17%), HIV was associated with stroke, but with a lower PAF than hypertension (5% vs 68%). There was no interaction between HIV and hypertension on stroke risk.

**Conclusions:**

In a population with high HIV prevalence, where stroke incidence is increasing, we have shown that HIV is an important risk factor. Early ART use in immunosuppressed patients poses an additional and potentially treatable stroke risk. Immune reconstitution inflammatory syndrome may be contributing to the disease mechanisms.

Across most of sub-Saharan Africa, the incidence of stroke is increasing.^1^ Much of this has been attributed to hypertension, but in countries such as Malawi and South Africa, a substantial proportion of stroke patients are young, and have a low prevalence of established risk factors such as hypertension, suggesting other factors may be important.^2,3^

It is postulated that HIV also predisposes to stroke.^4^ The virus may cause stroke directly (for example, through HIV-associated vasculopathy) or indirectly (through opportunistic infections).^4^ In addition, some drugs used in antiretroviral therapy (ART) for HIV are associated with metabolic syndromes, therefore potentially increasing stroke risk with prolonged use.^5^

Although several studies have looked at HIV and stroke, the effects of HIV infection, especially the role of ART, and its interaction with prevalent risk factors such as hypertension, particularly in African populations, remain uncertain.^4^ We therefore conducted a case-control study with prospective recruitment of cases and community controls, examining the role of HIV, its treatment, and its interaction with hypertension as risk factors for stroke in Malawian adults.

## METHODS

### Study site.

Malawi is located in southern Africa; the commercial capital is Blantyre. It is the 7th poorest country in the world. Life expectancy is 55 years. The national HIV prevalence is 10.3%, higher in comparison with other sub-Saharan African countries (5% in Tanzania [East Africa] and 3.2% in Nigeria [West Africa]).

Queen Elizabeth Central Hospital is the main hospital for the Blantyre district, as well as being the referral hospital for the southern region of Malawi. It has an estimated adult HIV prevalence of 18.5%.^6^ Approximately 1 million people reside in the Blantyre district.

### Study design.

Adult residents (age ≥18 years) of the Blantyre district who presented to the hospital within 7 days of the onset of symptoms who met the WHO case definition of stroke^7^ were recruited as study cases between February 2011 and April 2012. Participants were initially screened for eligibility by the study nurse using a standardized questionnaire, before a physician's review. Scans were performed on a GE (Milwaukee, WI) 0.35T Signa Ovation Excite MRI scanner within 7 days of admission. The images were reported by a local radiologist and subsequently reviewed by a neuroradiologist and an infectious diseases radiologist. Patients with recurrent stroke were eligible for inclusion, provided they had not already participated in the study.

**Figure 1:**
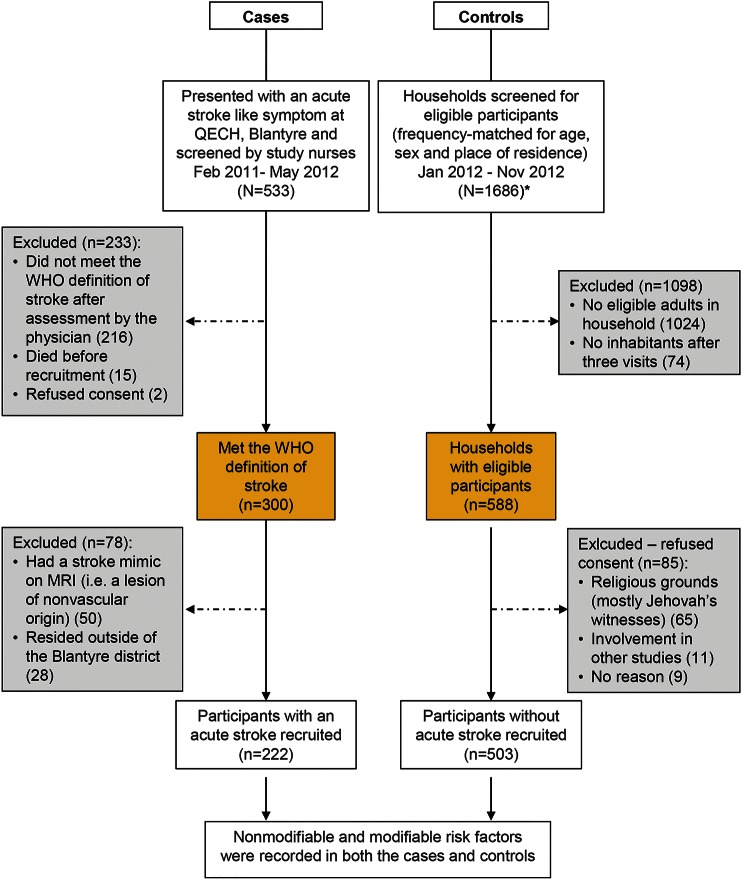
Flow diagram of case and control recruitment *Control selection: dwellings were visited and all eligible potential control participants were identified. If no one was home, dwellings were visited up to 3 times. Where multiple individuals were eligible, the oldest individual was recruited. If no eligible individual was identified, the next dwelling intersected was visited. Recruitment continued in each residential neighborhood until the prespecified numbers of individuals in each age and sex category had been met. QECH = Queen Elizabeth Central Hospital.

Population controls were recruited from the local population in predefined residential neighborhoods within the district of Blantyre between January and November 2012. Two community controls were selected at random for every case, using a modification of a previously described approach that selected an age/sex frequency-matched random sample of the population, with a geographical distribution in proportion to the population density.^8^ The distribution of age (5-year age bands), sex, and place of residence of the first 100 cases was used to guide stratified recruitment, frequency-matching for these factors (figure 1). Random starting points and direction were overlaid onto high-resolution satellite maps (March 2010 images: GeoEye-1/Eurimage SpA) using Google Earth pro software. Global Positioning System coordinates from the first dwelling intersected by any given randomly generated vector were recorded.

### Procedures.

For cases, the clinical subtype and severity of stroke were recorded using the Oxfordshire Community Stroke Project classification and NIH Stroke Scale (NIHSS) criteria.^9,10^ Ischemic and hemorrhagic stroke types were determined by MRI brain scan findings.

Demographic information, nonmodifiable risk factors (age, sex, socioeconomic status, and family history of stroke), and modifiable risk factors (HIV infection, HIV treatment and its duration, hypertension, diabetes, hypercholesterolemia, acute infection, smoking, alcohol use, pregnancy, and substance use [cannabis, cocaine, and heroin]) were recorded from cases and controls. Socioeconomic status was defined by education, occupation, place of residence, and housing type.^11^ Examination was performed to determine the waist-hip ratio, a marker of abdominal obesity, and blood pressure.^12^ Cases and controls were screened for diabetes, hypercholesterolemia, and HIV infection; if positive, the CD4^+^ count (CD4^+^ T-lymphocyte cell count; BD FACS Count System, Becton Dickinson, San Jose, CA) and HIV viral load (Hologic Commercial Kits, Sussex, UK) were measured.

### Definition of criteria used.

Stroke severity was classified as nonsevere or severe using the NIHSS.^9^ HIV diagnosis was made from 2 rapid tests in parallel (Unigold, Trinity Biotech, Wicklow, Ireland; Determine, Alere Medical, Philadelphia, PA; SD Bioline, Standard Diagnostics, Gyeonggi-do, Korea, was used as a tiebreaker). CD4^+^ count was classified as <200 cells/mm^3^, 200–349 cells/mm^3^, 350–500 cells/mm^3^, or >500 cells/mm^3^.^13^ To explore the possible role of immune reconstitution syndrome, a cutoff of 6 months was used to classify the duration of ART.^14^ The timing of ART was cross-checked with an electronic medical records system at Queen Elizabeth Central Hospital.^15^ The limit of sensitivity for HIV detection was <30 copies/mL. Blood pressure was recorded at day 0. Three sequential readings were recorded and an average taken. Hypertension was defined as a blood pressure >140/90 mm Hg or use of antihypertensive medication.^16^ Diabetes mellitus was defined as a nonfasting blood glucose of ≥11.1 mmol/L or use of glucose-lowering medication.^17^ Patients were classified as having hypercholesterolemia if they used lipid-lowering medication or had a nonfasting serum cholesterol concentration ≥6.2 mmol/L.^18^ Waist-hip ratio was calculated as tertiles from the control cohort.^12^ Recent infection was defined as a fever or treated infection within 14 days of the stroke (cases) or interview (controls).^19^

### Standard protocol approvals, registrations, and patient consents.

The study was approved by the Liverpool School of Tropical Medicine, United Kingdom, and the College of Medicine Research Ethics Committee, University of Malawi. All participants or guardians gave written informed consent. Individuals with newly diagnosed risk factors in the community were referred to an appropriate outpatient clinic.

### Statistical analysis.

The prevalence of potential risk factors was compared between cases and controls. Continuous variables were summarized using means and medians and compared using a Student independent-samples *t* test or Mann-Whitney *U* test as appropriate for the distribution properties of each variable.

A sample size of 750 individuals (250 case-control sets, each with 1 case and 2 controls) was estimated as providing 95% power to detect an odds ratio of 2 or greater for HIV prevalence, assuming that 18% of the control individuals would be found to have HIV infection.^6^

A conceptual framework was used to divide all other potential risk factors into proximal determinants that have direct effects on stroke and distal determinants that have indirect effects. Distal determinants were defined as age, sex, family history, socioeconomic status, and season. Proximal determinants were HIV status (also expanded or substituted by HIV treatment status and level of immunosuppression in some of the analysis), hypertension, diabetes, hypercholesterolemia, acute infection, abdominal obesity, and alcohol and substance use. The proximal determinants were evaluated individually (univariate model approach) after adjustment for age, sex, and urban location (variables used for frequency matching). Unconditional multivariate logistic regression models were then constructed based on this conceptual framework describing the hierarchical relationships among the matching, proximal, and distal determinants. The role of each distal determinant was further assessed by comparing the model with or without using the likelihood ratio test, subtracting variables from the model that were not significant.^20^ This was repeated after stratifying by age and type of stroke. Missing observations were included in the analysis by creating missing value categories. The findings from these models are reported as odds ratios with their 95% confidence intervals. All reported *p* values were 2-sided with a conventional 5% α level. Population attributable fraction (PAF) was calculated using methods defined by Greenland and Drescher.^21^ Interaction between the risk factors for stroke was assessed on an additive and multiplicative scale. Interaction on the additive scale was assessed by calculating the relative excess risk caused by interaction.^22,23^ Interaction on the multiplicative scale was assessed by comparing multiplicative models with and without an interaction term using the log-likelihood ratio test. The data were analyzed with STATA version 11.2.

## RESULTS

We screened 553 patients with suspected stroke and 1,686 households, to obtain 222 stroke cases and 503 controls (figure 1). An MRI brain scan was performed in 190/222 (86%) cases; 149 (78%) had findings consistent with an ischemic stroke and 41 (22%) a hemorrhagic stroke. Ischemic stroke subtype and severity are summarized in table 1; in the HIV population, the subtype and severity were similar in proportions to the HIV-negative cohort.

**Table 1:**
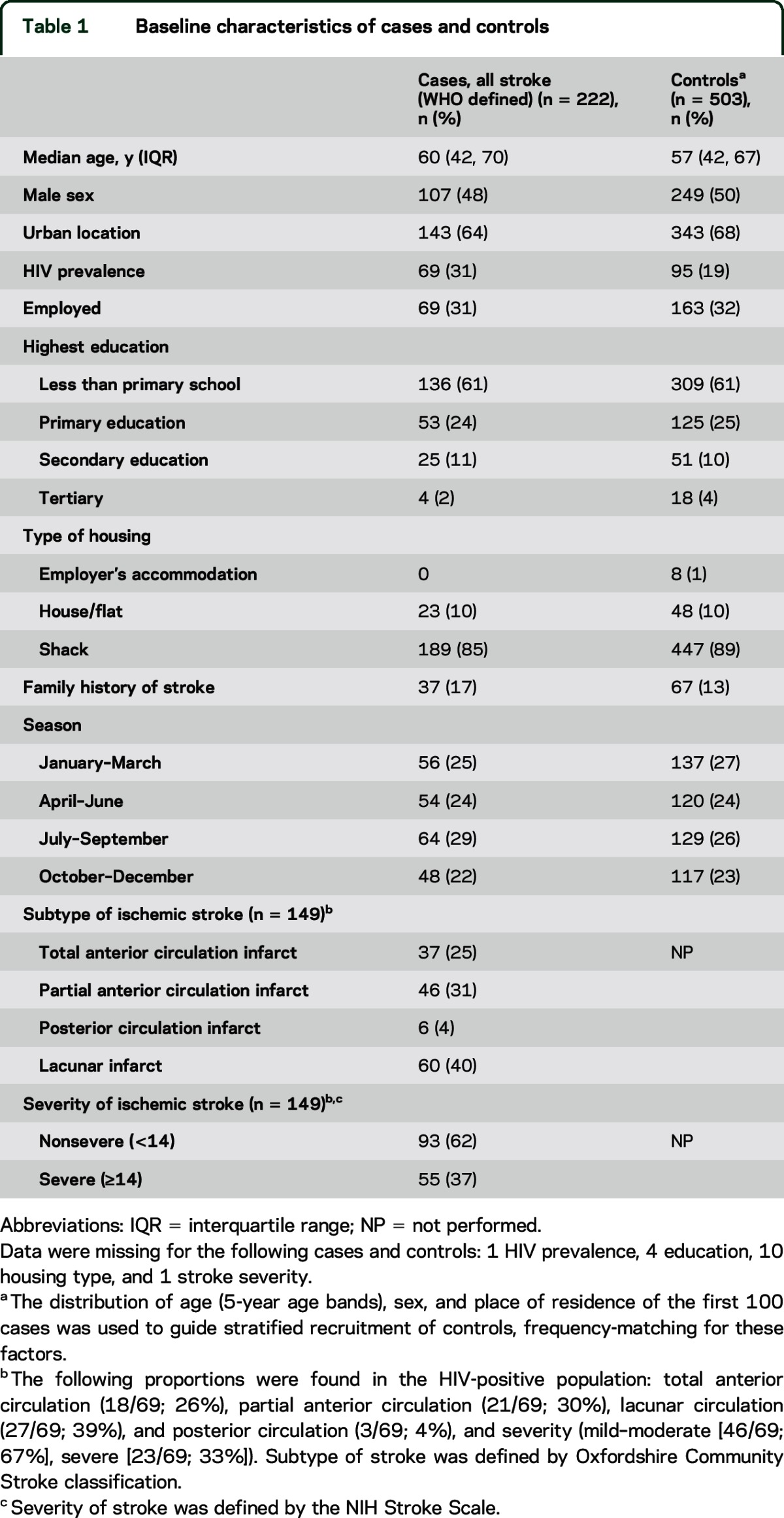
Baseline characteristics of cases and controls

Cases and controls were well matched for age, sex, socioeconomic status, and season of admission to the study (table 1). A sensitivity analysis was performed, excluding those without MRI (n = 32); the trends were consistent with those reported below.

### HIV and ART.

Stroke was strongly associated with HIV infection (adjusted odds ratio [aOR] 3.28). Timing of ART was important: having started ART in the previous 6 months (aOR 15.6) posed the greatest risk of stroke (table 2).

**Table 2:**
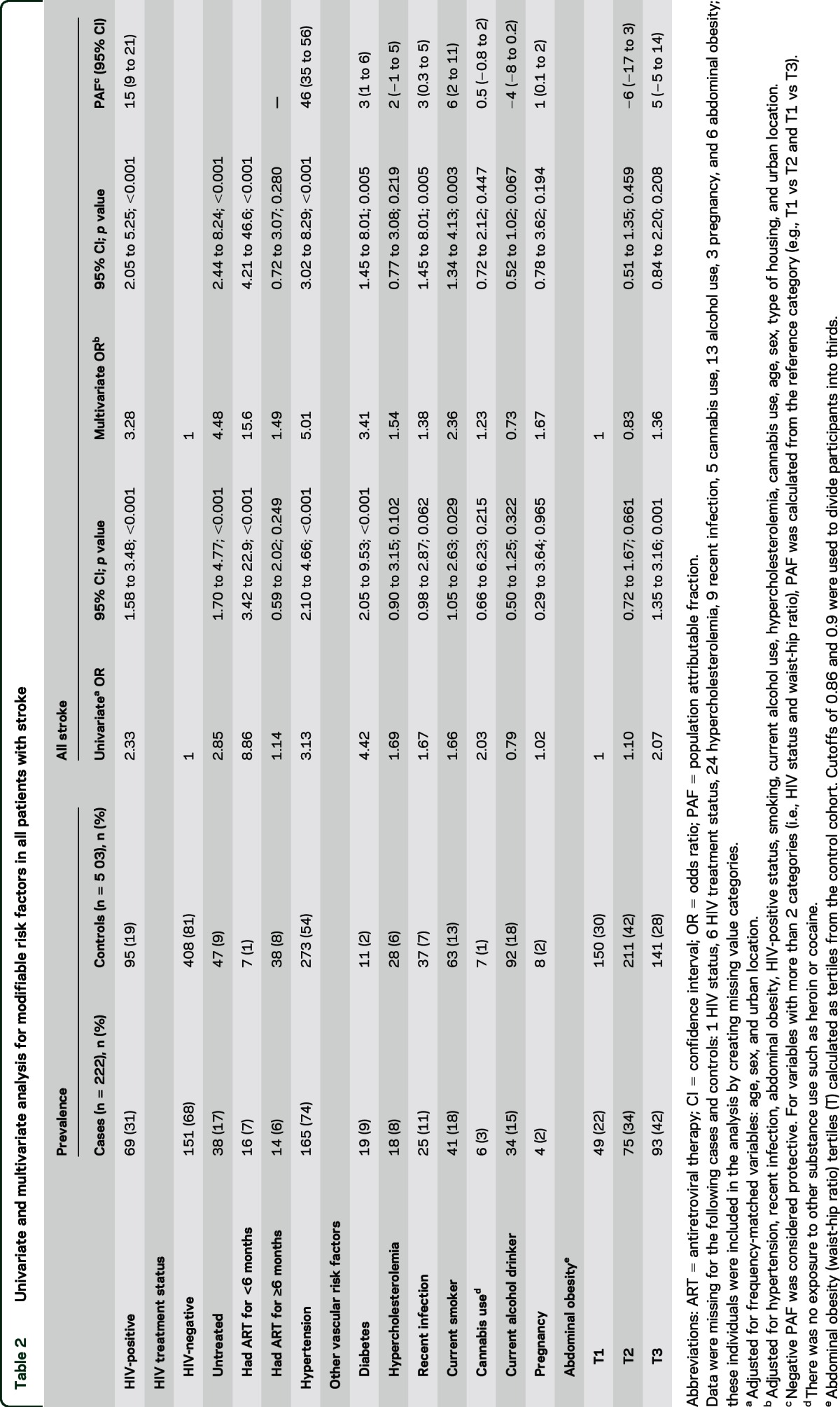
Univariate and multivariate analysis for modifiable risk factors in all patients with stroke

The association between HIV infection and stroke was more evident in younger individuals (≤45 years) (PAF 42%) compared to older individuals (PAF 6%; table 3). Although the prevalence of HIV was lower in the older participants (67% vs 18%), there was still a strong association with untreated HIV infection and with recently initiated ART (table 3). HIV infection was significantly associated with ischemic stroke (table e-1 on the *Neurology*® Web site at Neurology.org).

**Table 3:**
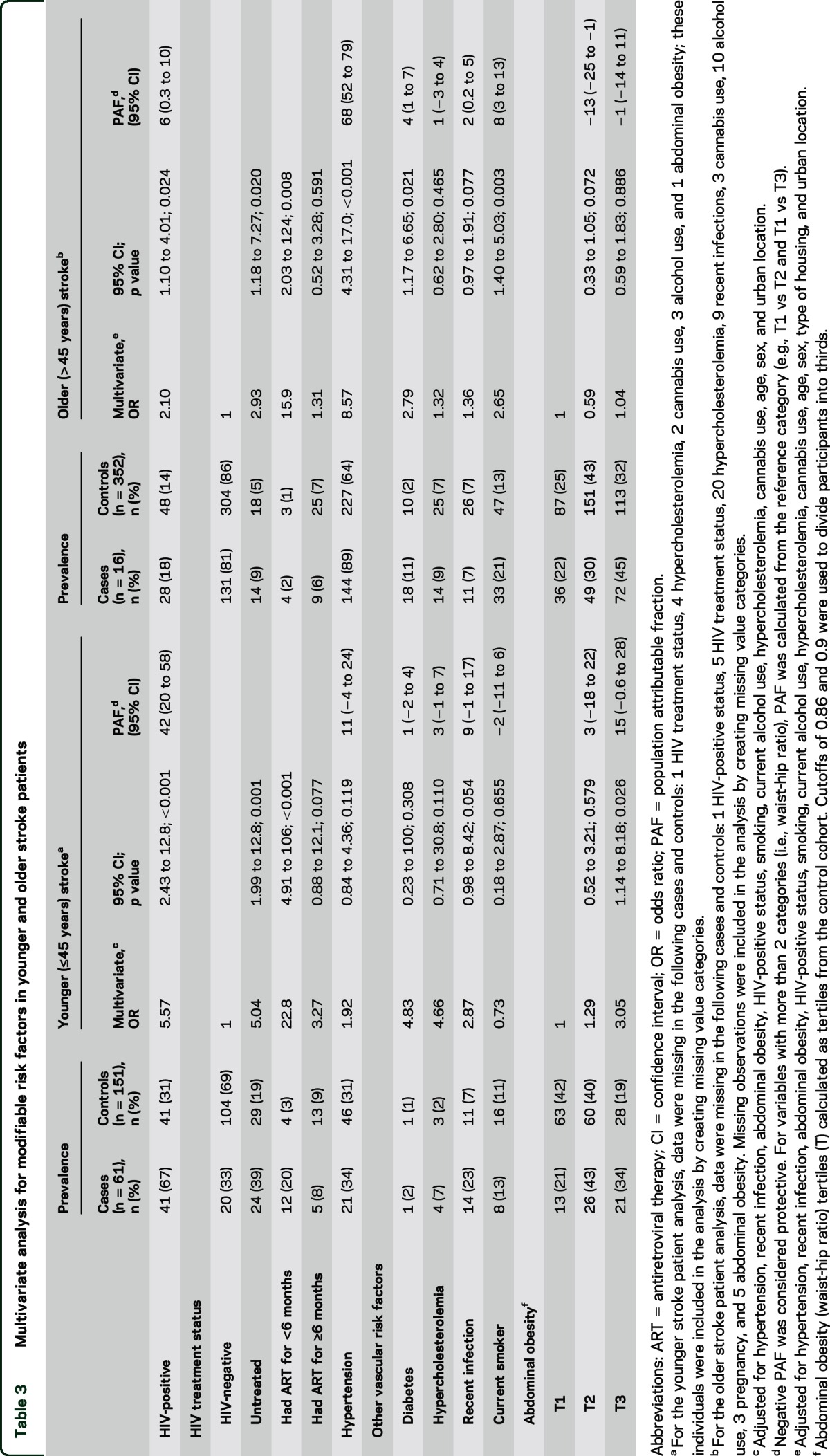
Multivariate analysis for modifiable risk factors in younger and older stroke patients

**Figure 2:**
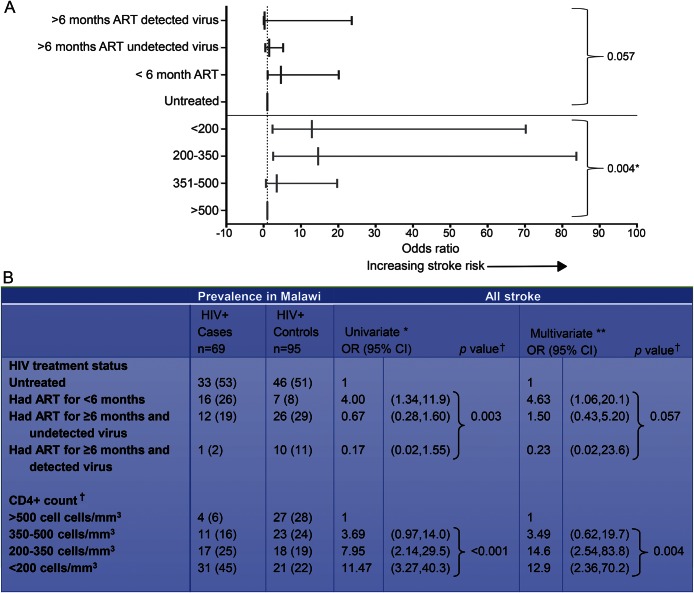
HIV treatment status, viral load, CD4^+^ count, and stroke risk (A) Multivariate analysis of HIV treatment status, CD4^+^ count, and stroke risk, represented graphically. (B) Univariate and multivariate analysis of HIV treatment status, CD4^+^ count, and stroke risk. Explores the association of HIV treatment status and stroke risk after adjusting for immunosuppression. *Adjusted for frequency-matched variables: age, sex, and urban location. **Adjusted for hypertension, recent infection, abdominal obesity, HIV treatment status, smoking, current alcohol use, CD4^+^ T-lymphocyte count, hypercholesterolemia, cannabis use, age, sex, type of housing, and urban location. †A combined *p* value was calculated using a likelihood ratio test for variables with >2 categories. Data were missing for the following cases and controls: 13 HIV treatment status (including HIV viral load data), 12 CD4^+^ T-lymphocyte cell count, 2 recent infection, 2 waist-hip ratio, 1 alcohol, 8 pregnant, 1 substance use. Missing observations were included in the analysis by creating missing value categories.

Thirty-one out of 69 (45%) cases and 21/95 (22%) controls met the definition of AIDS. We performed a subgroup analysis in the HIV-positive population. The reference group used was the HIV untreated cohort. We further subdivided the ≥6 months treatment group by the presence or absence of detectable HIV virus. We found that lower CD4^+^ count was a major risk factor for HIV-related stroke risk (figure 2). Furthermore, the CD4^+^ count was significantly lower among those starting ART in the first 6 months (92 vs 375 cells/mm^3^; *p* = 0.004) (table e-2). There was no association with HIV viral load and stroke (table e-2).

First-line therapy for HIV infection was stavudine (d4T), lamivudine (3TC), and nevirapine (NVP) and alternative first line was zidovudine (AZT), 3TC, and NVP. The prevalence of the stavudine-based regimen in cases and controls was 23 and 13 (64% and 36%; *p* < 0.001), respectively. No patients were on protease inhibitors.

### Hypertension.

Hypertension was strongly associated with stroke overall (aOR 5.01; table 2). The risk was stronger for hemorrhagic stroke (aOR 9.35; table e-1). The effect of hypertension was more evident in those >45 years old than in those ≤45 years old (table 3). Of the 438 participants with hypertension, only 118 (27%) were on treatment.

### Interaction of HIV and hypertension.

The combined aOR between HIV infection and hypertension for stroke was 13.63 (6.30, 29.45; table e-3), with no evidence of effect modification between these 2 risk factors and stroke risk (table e-3).

### Other vascular risk factors.

Diabetes was associated with a small proportion of strokes overall (aOR 3.41; table 2). Smoking (PAF 6%) and recent infection (PAF 3%) were also associated with stroke (table 2). There was no significant association between hypercholesterolemia and stroke (tables 2 and 3 and e-1); all participants were untreated. Cannabis was not a risk factor for stroke. None of our study participants used cocaine or amphetamines.

## DISCUSSION

This case-control study showed that HIV infection is an independent risk factor for stroke in Malawian adults. Notably, we also found that for patients who had started standard HIV treatment in the previous 6 months, the risk of stroke was even higher (aOR 15.6). Although hypertension was the leading risk factor in this population overall (PAF 46%), HIV infection and its treatment were the second most important risk factors (PAF 15%), and had the highest population attributable risk in younger patients (PAF 42%).

Most previous studies of HIV infection and stroke have been retrospective,^4^ but one recent prospective study reported the importance of HIV as a risk factor in East Africa^24^; however, this was limited by a low number of patients having brain imaging to confirm the stroke, and a low number having HIV testing,^25^ both reducing the power to investigate any relationship among the nature of stroke, timing of ART, and the interaction between hypertension and HIV infection. Our study, with well-defined cases, carefully selected population controls, and 99% ascertainment of HIV status, provides the clearest evidence yet that HIV is indeed an important risk factor for stroke. We found that HIV infection was associated with ischemic but not hemorrhagic stroke. A variety of mechanisms might be implicated: HIV infection is known to cause endothelial dysfunction, resulting in a vasculopathy that can manifest in several forms (e.g., accelerated atherosclerosis and small vessel disease); in addition, opportunistic infections such as VZV, which are more common in HIV infection, can also disrupt the vascular endothelium.^4^ Further evaluation of infections is beyond the scope of this article.

Importantly, we found that the risk of stroke was much higher in the first 6 months after starting ART. This clinical deterioration suggests a contribution to risk from an immune reconstitution inflammatory syndrome (IRIS)–like process.^14^

In HIV infection, IRIS is thought to be the consequence of an overwhelming pathogen-specific, cell-mediated immune response, arising either through unmasking of an occult infection or a paradoxical deterioration following HIV treatment.^14^ In the latter, the recovering immune response is thought to target persisting pathogen-derived antigens or, possibly, self-antigens, causing tissue damage.^14^ In the case of stroke, this immune process could target antigens arising from HIV-mediated tissue damage or HIV itself. We showed that stroke risk associated with ART in the first 6 months (and in the untreated cohort) was associated with immunosuppression. In contrast, those on longer-term treatment had a much higher CD4^+^ count, possibly explaining the reduced stroke risk in this group. Immunosuppression is a recognized risk factor for IRIS and thus a plausible mechanism of stroke among those starting ART. There is also the possibility of other simultaneous or independent mechanisms (beyond immunosuppression) occurring during this phase, such as stavudine toxicity and poor ART adherence; however, this needs to be further characterized.^26,27^ Because our numbers were limited in the early ART cohort, the results should be interpreted with caution. However, insights from the large prospective Data Collection on Adverse Events of Anti-HIV Drugs (D:A:D) study support our findings.^28^ In this study, there was an increased risk of cardiovascular and cerebrovascular disease events for 4 years after starting ART; although it was not highlighted at the time, the data indicate the rate of vascular events was greatest in the first year of follow-up, consistent with a temporal relationship to initiation of ART (figure e-1).^28^

Higher CD4^+^ counts (a function of ART) were associated with reduced stroke risk, suggesting that HIV treatment is indeed beneficial overall. However, the stroke risk in the immunosuppressed early ART group needs further investigation; a better understanding may point to empirical antimicrobial treatment of occult infection or initiation of anti-inflammatory agents at the time of starting ART.

There was no statistically significant additive or multiplicative interaction between hypertension and HIV infection on stroke risk. Although our study was underpowered to fully address this, our findings are consistent with those of a large US cohort study investigating interactions among HIV, hypertension, and cardiovascular disease: in individuals with acute myocardial infarction, no such interaction was seen.^29^ In Malawi, we found hypertension was an important risk factor in older, but not younger, stroke patients. This finding is different from the multinational interstroke study, which attributed most young strokes in low- and middle-income countries to hypertension; in that study, only one fifth of the patients were from sub-Saharan Africa, and most of these were from wealthier African countries where hypertension, diabetes, and hypercholesterolemia are likely to occur with higher prevalence than in Malawi.^12,30,31^

Our study had several limitations. Recall bias cannot be excluded, but the prospective recruitment of cases and objective measurement of the important risk factors has considerably reduced this possibility. Although blood pressure measurement in the acute phase of a stroke could potentially overestimate stroke risk, our findings were consistent with a stroke study in East Africa; this study measured blood pressure on day 7.^24^ The low prevalence of high cholesterol in Malawi or not measuring the more sensitive low-density lipoprotein could explain the lack of association between cholesterol and stroke in our study.^30^ The absence of an association between HIV and hemorrhagic stroke could be due to insufficient power. Because the hospital prevalence of HIV infection was much higher (70%)^32^ than in the community (18%), using hospital controls would have distorted the stroke risk seen with HIV infection. We therefore opted for population controls to avoid this bias. Arguably, by using population controls, the risk seen with hypertension could have been overestimated but the result of this risk factor was consistent with those from similar settings.^24^ In the community, the refusal rate for entering the study was low (14%), minimizing selection bias. The HIV prevalence in our control cohort was consistent with a previous study in this setting,^6^ supporting the validity of our control selection process. Because age, sex, and urban location are important confounders of stroke and HIV infection, we adopted a stratified approach for these variables. This was especially important in our setting because the age of stroke patients was considerably greater than the median age of Blantyre residents. Although this stratification minimized selection bias, it limited our ability to comment on these factors or their effect modification on stroke risk. The PAF was not affected by underlying age structure and the formula can be calculated using the distribution of risk factors among the cases alone, independent of the control population.^33^ Hospital recruitment of cases could have led to selection bias, missing out on milder strokes or patients who may have died in the community.^34^ However, a study in East Africa used a hot pursuit approach and verbal autopsy to capture these cases; they described similar risk factor profiles to ours. Therefore, we suspect that such potential biases are unlikely to have affected our risk factor analysis.^24^

HIV infection is an important risk factor for stroke in Malawi, especially ischemic stroke in young people. Importantly, there was a high risk of stroke in the first 6 months after starting ART; this was largely related to immunosuppression. While ART is necessary for improved HIV related outcomes overall, the association between early ART use and stroke events poses an additional (and potentially treatable) risk. How to address this is something that the research community will need to consider in sub-Saharan Africa and beyond.

## Supplementary Material

Data Supplement

Accompanying Editorial

## GLOSSARY

3TC: lamivudine
aOR: adjusted odds ratio
ART: antiretroviral therapy
AZT: zidovudine
d4T: stavudine
IRIS: immune reconstitution inflammatory syndrome
NIHSS: NIH Stroke Scale
NVP: nevirapine
PAF: population attributable fraction
