# Supplementary material for: HIV, antiretroviral treatment, hypertension, and stroke in Malawian adults: A case-control study
Source: Neurology. 2016 Jan 26;86(4):324–33. doi: 10.1212/WNL.0000000000002278 (PMC4776088; doi:10.1212/WNL.0000000000002278)
Supplement: Data Supplement [file supp_86_4_324__index.html]

Data Supplement 

# HIV, antiretroviral treatment, hypertension, and stroke in Malawian adults

## Data Supplement

Three tables and one figure; four Microsoft Word files.

**Neurology® data supplements are not copyedited before publication. Published editorials and translations have been copyedited.  
 © 2016 American Academy of Neurology.  
  
 Files in this Data Supplement:**

- Table e-1 - Microsoft Word file
- Table e-2 - Microsoft Word file
- Table e-3 - Microsoft Word file
- Figure e-1 - Microsoft Word file
