# Supplementary material for: HIV, antiretroviral treatment, hypertension, and stroke in Malawian adults: A case-control study
Source: Neurology. 2016 Jan 26;86(4):324–33. doi: 10.1212/WNL.0000000000002278 (PMC4776088; doi:10.1212/WNL.0000000000002278)
Supplement: Data Supplement [file supp_WNL.0000000000002278_Table_e-1.docx]

| Table e-1 Multivariate analysis for modifiable risk factors in ischemic and hemorrhagic stroke patients | | | | | | | | |
| --- | --- | --- | --- | --- | --- | --- | --- | --- |
|  | **Prevalence** | | **Ischemic stroke *** | | **Prevalence** | | **Hemorrhagic stroke **** | |
|  | **Cases n=149 (%)** | **Controls**  **n=503 (%)** | **Adjusted OR (95% CI) p value** | | **Cases**  **n=41 (%)** | **Controls**  **n=503 (%)** | **Adjusted OR (95% CI) p value** | |
| **HIV positive status** | 57 (38) | 95 (19) | 4.36 | (2.58,7.36) <0.001 | 8 (20) | 95 (19) | 1.79 | (0.63,5.05) 0.274 |
| **HIV treatment status^‡^**  HIV negative  Untreated  Had ART for <6 months  Had ART for ≥6 months | 92 (62)  34 (23)  13 (9)  9 (6) | 408 (81)  47 (9)  7 (1)  38 (8) | 1  6.31  20.2  1.56 | (3.27,12.1) <0.001  (6.31,64.4) <0.001  (0.65,3.69) 0.314 | 32(78)  3 (7)  2 (5)  3 (7) | 408 (81)  47 (9)  7 (1)  38 (8) | 1  2.27  5.17  1.42 | (0.45,11.4) 0.410  (0.56,48.0) 0.119  (0.36,5.66) 0.200 |
| **Hypertension^¶^** | 103 (69) | 273 (54) | 3.66 | (2.15,6.22) <0.001 | 35 (85) | 273 (54) | 9.35 | (2.89,30.3) <0.001 |
| **Other vascular risk factors**  **Diabetes**  **Hypercholesterolemia**  **Recent infection**  **Current smoker^¥^**  ***Abdominal obesity*** ∞  T1  T2  T3 | 12 (8)  12 (8)  18(12)  30 (20)  29(19)  52 (35)  67 (45) | 11 (2)  28 (6)  37 (7)  63 (13)  150 (30)  211 (42)  141 (28) | 3.70  1.65  1.53  2.48  1  0.93  2.01 | (1.42,9.63) 0.007  (0.76,3.61) 0.208  (1.07,2.19) 0.020  (1.34,4.61) 0.004  (0.53,1.61) 0.788  (1.15,3.49) 0.014 | 1 (2)  5 (12)  6 (15)  6 (15)  15 (36)  9 (22)  17 (41) | 11 (2)  28 (5)  37 (7)  63 (13)  150 (30)  211 (42)  141 (28) | 0.33  1.62  2.02  3.12  1  0.36  0.73 | (0.03,3.59) 0.365  (0.47,5.57) 0.441  (1.28,3.19) 0.002  (0.85,11.5) 0.087  (0.13,0.97) 0.044  (0.30,1.80) 0.500 |

*****Adjusted for hypertension, recent infection, abdominal obesity, HIV positive status, smoking, current alcohol drinker, hypercholesterolemia, cannabis use, age, sex, type of housing and urban location.

******Adjusted for hypertension, recent infection, abdominal obesity, HIV positive status, smoking, current alcohol drinker, hypercholesterolemia, cannabis use, age, sex, type of housing, level of schooling and urban location.

**†**For ischemic stroke analysis - data were missing in the following cases and controls: One HIV positive status, 4 HIV treatment status, 18 hypercholesterolemia, 6 recent infection and 2 abdominal obesity.

**‡**For hemorrhagic stroke - data were missing in the following cases and controls: Four for HIV treatment status, 10 hypercholesterolemia, 5 recent infections and 1 abdominal obesity. Missing observations were included in the analysis by creating missing value categories.

∞Waist-to-hip ratio tertiles (T) was calculated as tertiles from the control cohort. Cutoffs of 0.86 and 0.9 were used to divide participants into thirds.
