# Supplementary material for: HIV, antiretroviral treatment, hypertension, and stroke in Malawian adults: A case-control study
Source: Neurology. 2016 Jan 26;86(4):324–33. doi: 10.1212/WNL.0000000000002278 (PMC4776088; doi:10.1212/WNL.0000000000002278)
Supplement: Data Supplement [file supp_WNL.0000000000002278_Table_e-2.docx]

| **Table e-2 CD4+ T-lymphocyte count and HIV viral load in cases and controls** | | | | |
| --- | --- | --- | --- | --- |
|  | | **Cases** | **Controls** | **P value** |
| **Overall** | N  Median HIV VL log10 copies/ml  Median CD4 count (cells/mm3) | 62  2.41 (0,4.55)  190 (74,338) | 89  2.88 (0,5.00)  375 (200,533) | 0.397  <0.001* |
|  | Untreated | 175 (66,322)^‡^ | 336 (166,513) | 0.003* |
| **Median CD4 count by HIV treatment status** | Had ART for <6 months | 92 (50,218) | 375 (230,454) | 0.004* |
|  | Had ART for ≥6 months and undetected HIV virus | 318 (278,460) | 445 (353,714) | 0.135 |
|  | Had ART for ≥6 months and detected HIV virus | 411(411,411) | 385(236,483) | 1.00 |
| HIV VL HIV viral load  CD4+ count CD4+ T-lymphocyte count  ‡Median (Interquartile range) | | | |  |
