# Supplementary material for: HIV, antiretroviral treatment, hypertension, and stroke in Malawian adults: A case-control study
Source: Neurology. 2016 Jan 26;86(4):324–33. doi: 10.1212/WNL.0000000000002278 (PMC4776088; doi:10.1212/WNL.0000000000002278)
Supplement: Data Supplement [file supp_WNL.0000000000002278_Table_e-3.docx]

| **Table e-3 The combined effects of HIV infection and Hypertension on the risk of stroke** | | | | |
| --- | --- | --- | --- | --- |
|  | **Hypertension negative** | | **Hypertension positive** | |
|  | Cases/controls (N) | aOR (95% CI) | Case/control (N) | aOR (95% CI) |
| **HIV  negative** | 21/162 | 1 | 131/246 | 5.33 (2.93,9.69) |
| **HIV positive** | 35/68 | 4.41 (2.23,8.75) | 34/27 | 13.63(6.30,29.45) |

Relative excess risk due to interaction (RERI) 3.51 (-3.22,10.26)

Multiplicative risk due to interaction 0.58 (0.24,1.42)

OR is adjusted recent infection, abdominal obesity, smoking, current alcohol drinker, hypercholesterolemia, cannabis use, age, sex, type of housing  and urban location
