# Supplementary material for: HIV, antiretroviral treatment, hypertension, and stroke in Malawian adults: A case-control study
Source: Neurology. 2016 Jan 26;86(4):324–33. doi: 10.1212/WNL.0000000000002278 (PMC4776088; doi:10.1212/WNL.0000000000002278)
Supplement: Data Supplement [file supp_WNL.0000000000002278_Figure_e-1.docx]

**Figure e-1** **Incidence of stroke and myocardial infarction following ART initiation in a US population.**

**
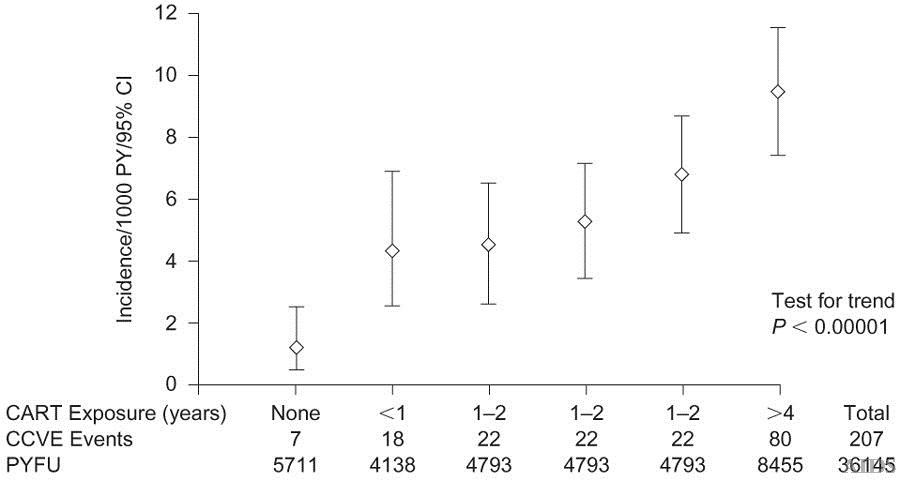
**

Shows the incidence of vascular events after combination antiretroviral therapy (ART) initiation in a US population. This was assessed according to the cumulative duration of ART since initiation, stratified in 1-year intervals from 0–4 years, an interval of more than 4 years exposure and an interval for no exposure. The red box highlights the highest rate of vascular events was within the first year of the 0-4 years interval. P-value for trends: < 0.00001. Reproduced from D’Arminio and colleagues, by permission of Lippincott Williams & Wilkins.

PY, person-years; CI, confidence interval; (C)ART, combination antiretroviral therapy; CCVE, cardio- and cerebro-vascular events
