# Supplementary material for: HIV, antiretroviral treatment, hypertension, and stroke in Malawian adults: A case-control study
Source: Neurology. 2016 Jan 26;86(4):324–33. doi: 10.1212/WNL.0000000000002278 (PMC4776088; doi:10.1212/WNL.0000000000002278)
Supplement: Accompanying Editorial [file supp_WNL.0000000000002278_316.pdf]

EDITORIAL

The merging burden of HIV infection and stroke in the developing world

Réza Behrouz, DO
Rebecca F. Gottesman, MD, PhD

Correspondence to Dr. Behrouz: behrouz@uthscsa.edu

Neurology® 2016;86:316–317

According to the WHO, at the end of 2014, the majority of the approximately 36.9 million people living with HIV/AIDS resided in low- to middle-income countries in sub-Saharan Africa.[1] The same report indicated that cases in sub-Saharan Africa account for almost 70% of the global total of new HIV infections.[1] The burden of stroke in developing countries parallels that of HIV/AIDS. Approximately 80% of people who have had a stroke live in low- to middle-income nations.[2] Over the past 4 decades, stroke incidence in these countries has increased by more than 100%.[3] In developing nations, HIV/AIDS and stroke are not mutually exclusive. Growing evidence suggests that HIV infection independently increases the risk for stroke.[4,5] Given the high prevalence of HIV/AIDS in sub-Saharan Africa, HIV infection may be the cause for a measureable subset of strokes in this region. However, incomplete disease surveillance and record-keeping make studying the relationship between HIV/AIDS and stroke difficult, especially in low-income nations.

In this issue of *Neurology*®, Benjamin et al.[6] describe a strong association between HIV and stroke in a prospective, case-control, hospital-based study in Malawi, a low-income country in sub-Saharan Africa with a high prevalence of HIV infection. The investigators aimed to determine the risk of stroke independently attributable to HIV, antiretroviral therapy (ART), and hypertension. HIV infection was the predominant risk factor for stroke in individuals aged ≤45 years, whereas hypertension surpassed HIV infection (adjusted odds ratio 8.57 vs 2.10) in older adults. Furthermore, lower relative $CD4^+$ lymphocyte count in untreated HIV and HIV infection with recent initiation of ART also were associated with increased risk of stroke.

This study provides critical information in the evaluation of HIV infection and stroke in Africa, particularly given the likely obstacles to conducting longitudinal analyses in developing nations. It supports prior reports that HIV infection is an independent risk factor for stroke, and demonstrates that ART—early in its use—further contributes to this risk. By evaluating hypertension as another important risk factor, the authors parse out the relative importance of these 2 major risk factors in this population, with the novel finding that hypertension is a more important risk factor than HIV in older Malawian adults. The increased risk of stroke in younger HIV-infected patients found by this study is consistent with prior reports.[4]

The study has limitations, especially with regard to generalizability. The investigation was conducted in an extremely poor country with a paucity of resources including limited access to highly active or combination ART. The finding by the authors of increased risk of stroke early in the course of ART is only valid for nucleoside and non-nucleoside reverse transcriptase inhibitors since none of the patients in this study were on protease inhibitors, a class of drugs strongly associated with hyperlipidemia and insulin resistance.[7] Furthermore, those starting ART in the first 6 months had very low $CD4^+$ counts, suggesting that many were in advanced stages of AIDS. Because of the observational nature of these data, subjects with recent initiation of ART may have differed in other substantive ways from the group with longer-term treatment (including compliance). Therefore, the adverse impact of ART on the risk of stroke should be considered in the context of duration, severity, and the stage of HIV infection. The large confidence intervals associated with the risk of stroke in patients on ART for less than 6 months suggest limited power to allow unequivocal conclusions. Similar to concerns about generalizability of the patient population, controls were selected from the community, not from a group of hospitalized individuals. Stroke patients in this study may therefore have had other medical comorbidities or infections that could not be evaluated by the investigators. These concomitant conditions might not be found in the community controls, yet could explain the apparent associations. Moreover, information about specific HIV-associated opportunistic or coinfections was not available in this cohort.

The study's finding that decreasing $CD4^+$ lymphocyte count is associated with an increasing risk of stroke may further support a role of underlying

See page 324

From the Department of Neurology (R.B.), School of Medicine, University of Texas Health Science Center, San Antonio; and the Department of Neurology (R.F.G.), Johns Hopkins University School of Medicine, Baltimore, MD.

Go to Neurology.org for full disclosures. Funding information and disclosures deemed relevant by the authors, if any, are provided at the end of the editorial.

© 2015 American Academy of Neurology

© 2015 American Academy of Neurology. Unauthorized reproduction of this article is prohibited.

HIV infection in the stroke cases. Interestingly, the authors did not find a similar association for increasing viral load. It remains unclear which marker better estimates this risk, given that others have described the opposite pattern; that is, viral load, not $CD4^+$ count, associated with an increased risk of stroke.[4] Although the extent of immunodeficiency represented by $CD4^+$ lymphocyte count certainly influences the risk of stroke, a direct viro-toxic effect of HIV on the arteries and its role in accelerated atherosclerosis cannot be undermined.[8,9] The theory that vascular risk might directly relate to viral titers has not been studied systematically. Still, in developing countries with inherently limited resources, routine performance of both tests may not prove a cost-effective strategy.[10] Determination of the predictive value of each test in terms of stroke and cardiovascular risk needs further investigation.

We commend Benjamin et al. in meticulously conducting a prospective study of a reasonable size to answer a complex question. Their investigation included a cohort of HIV-infected individuals not on ART, an elusive yet ideal population for delineating the risk of stroke directly related to HIV infection. It also further supports the importance of prevention and treatment of standard vascular risk factors (in this case, hypertension), particularly in older adults, even in low-income countries with high rates of HIV disease.

STUDY FUNDING

No targeted funding reported.

DISCLOSURE

R. Behrouz and R. Gottesman report no disclosures relevant to the manuscript. Go to Neurology.org for full disclosures.

REFERENCES

1. World Health Organization. 2014 Fact Sheet for HIV/AIDS. Available at: http://www.who.int/mediacentre/factsheets/fs360/en/. Accessed August 20, 2015.
2. Truelsen T, Heuschmann PU, Bonita R, et al. Standard method for developing stroke registers in low-income and middle income countries: experiences from a feasibility study of a stepwise approach to stroke surveillance (STEPS Stroke). Lancet Neurol 2007;6:134–139.
3. Feigin VL, Lawes CM, Bennett DA, Barker-Collo SL, Parag V. Worldwide stroke incidence and early case fatality reported in 56 population-based studies: a systematic review. Lancet Neurol 2009;8:355–369.
4. Chow FC, Regan S, Feske S, Meigs JB, Grinspoon SK, Triant VA. Comparison of ischemic stroke incidence in HIV-infected and non-HIV-infected patients in a US health care system. J Acquir Immune Defic Syndr 2012;60:351–358.
5. Benjamin LA, Bryer A, Emsley HCA, Khoo S, Solomon T, Connor MD. HIV infection and stroke: current perspectives and future directions. Lancet Neurol 2012;11:878–890.
6. Benjamin LA, Corbett EL, Connor MD, et al. HIV, antiretroviral treatment, hypertension, and stroke in Malawian adults: a case-control study. Neurology 2016;86:324–333.
7. Carr A, Samaras K, Burton S, et al. A syndrome of peripheral lipodystrophy, hyperlipidaemia and insulin resistance in patients receiving HIV protease inhibitors. AIDS 1998;12:F51–F58.
8. Maggi P, Perilli F, Lillo A, et al. An ultrasound-based comparative study on carotid plaques in HIV-positive patients vs. atherosclerotic and arteritis patients: atherosclerotic or inflammatory lesions? Coron Artery Dis 2007;18:23–29.
9. Ho JE, Hsue PY. Cardiovascular manifestations of HIV infection. Heart 2009;95:1193–1202.
10. Brown ER, Otieno P, Mbori-Ngacha DA, et al. Comparison of CD4 cell count, viral load and other markers for the predication of mortality among HIV-1–infected Kenyan pregnant women. J Infect Dis 2008;199:1292–3100.

© 2015 American Academy of Neurology. Unauthorized reproduction of this article is prohibited.
